# Digital Global Recruitment for Women’s Health Research: Cross-sectional Study

**DOI:** 10.2196/39046

**Published:** 2022-09-14

**Authors:** Erika Rodriguez, Komal Peer, Victoria Fruh, Kaitlyn James, Anna Williams, Alexis de Figueiredo Veiga, Michael R Winter, Amanda Shea, Ann Aschengrau, Kevin J Lane, Shruthi Mahalingaiah

**Affiliations:** 1 Department of Environmental Health Harvard TH Chan School of Public Health Boston, MA United States; 2 Deborah Kelly Center for Outcomes Research Department of Obstetrics and Gynecology Massachusetts General Hospital Boston, MA United States; 3 Biostatistics and Epidemiology Data Analytics Center Boston University School of Public Health Boston, MA United States; 4 Clue by BioWink GmbH Berlin Germany; 5 Department of Epidemiology Boston University School of Public Health Boston, MA United States; 6 Department of Environmental Health Boston University School of Public Health Boston, MA United States; 7 Division of Reproductive Endocrinology and Infertility Department of Obstetrics and Gynecology Massachusetts General Hospital Boston, MA United States

**Keywords:** digital recruitment, internet, menstrual tracking app, menstrual, menstruation, reproductive health, reproduction, mobile health, menstrual health, mHealth, women's health, Facebook, social media, epidemiology research, in-app message, tracking app, health application, health app, eHealth, digital health, health technology, ovulation, recruit, attrition, research subject, participation, participant

## Abstract

**Background:**

With the increased popularity of mobile menstrual tracking apps and boosted Facebook posts, there is a unique opportunity to recruit research study participants from across the globe via these modalities to evaluate women’s health. However, no studies to date have assessed the feasibility of using these recruitment sources for epidemiological research on ovulation and menstruation.

**Objective:**

The objective of this study was to assess the feasibility of recruiting a diverse sample of women to an epidemiological study of ovulation and menstruation (OM) health (OM Global Health Study) using digital recruitment sources. The feasibility and diversity were assessed via click and participation rates, geographic location, BMI, smoking status, and other demographic information.

**Methods:**

Participants were actively recruited via in-app messages using the menstrual tracking app Clue (BioWink GmbH) and a boosted Facebook post by DivaCup (Diva International Inc.). Other passive recruitment methods also took place throughout the recruitment period (eg, email communications, blogs, other social media). The proportion of participants who visited the study website after viewing and clicking the hypertext link (click rates) in the in-app messages and boosted Facebook post and the proportion of participants who completed the surveys per the number of completed consent and eligibility screeners (participation rates) were used to quantify the success of recruiting participants to the study website and study survey completion, respectively. Survey completion was defined as finishing the pregnancy and birth history section of the OM Global Health Study questionnaire.

**Results:**

The recruitment period was from February 27, 2018, through January 24, 2020. In-app messages and the boosted Facebook post were seen by 104,000 and 21,400 people, respectively. Overall, 215 participants started the OM Global Health Study survey, of which 140 (65.1%), 39 (18.1%), and 36 (16.8%) participants were recruited via the app, the boosted Facebook post, and other passive recruitment methods, respectively. The click rate via the app was 18.9% (19,700 clicks/104,000 ad views) and 1.6% via the boosted Facebook post (340 clicks/21,400 ad views.) The overall participation rate was 44.6% (198/444), and the average participant age was 21.8 (SD 6.1) years. In terms of geographic and racial/ethnic diversity, 91 (44.2%) of the participants resided outside the United States and 147 (70.7%) identified as non-Hispanic White. In-app recruitment produced the most geographically diverse stream, with 44 (32.8%) of the 134 participants in Europe, 77 (57.5%) in North America, and 13 (9.8%) in other parts of the world. Both human error and nonhuman procedural breakdowns occurred during the recruitment process, including a computer programming error related to age eligibility and a hacking attempt by an internet bot.

**Conclusions:**

In-app messages using the menstrual tracking app Clue were the most successful method for recruiting participants from many geographic regions and producing the greatest numbers of started and completed surveys. This study demonstrates the utility of digital recruitment to enroll participants from diverse geographic locations and provides some lessons to avoid technical recruitment errors in future digital recruitment strategies for epidemiological research.

## Introduction

Increased access to the internet via smartphones allows individuals to obtain information to better understand their menstrual cycles via social media, content-specific blogs, and mobile health apps. As of 2021, 93% of individuals in the United States use the internet. This usage is consistent across racial/ethnic groups, with 91% of Black Americans, 93% of White Americans, and 95% of Hispanic Americans using the internet [1]. In January 2018, 95% of Americans owned a mobile phone and 77% of those owned a smartphone. These numbers have steadily increased. In February 2021, 97% of Americans owned a mobile phone and 85% owned a smartphone [2]. Not only has access to these devices increased, but as of 2020, there were more than 300 reproductive health apps in the Apple App and Google Play Stores largely targeting women of childbearing age [3]. In this paper, we refer to individuals who menstruate as “women,” but we acknowledge that not all of those who menstruate identify as women. In 2019, the 5 most popular mobile menstrual tracking apps, according to obstetricians/gynecologists interviewed for an article in *Women’s Health*, were Clue (BioWink GmbH), Flo (Flo Health Inc.), Ovia Fertility (Ovia Health), Eve by Glow (Glow Inc), and MagicGirl [4]. Recruitment via mobile menstrual tracking apps presents unique opportunities to advance epidemiological research on menstruation [5,6].

Researchers are beginning to take advantage of health apps and online platforms, including blogs, for recruitment of study participants [7]. In particular, the use of boosted Facebook posts for recruitment to health research is increasing in popularity, and recent studies have examined their use to recruit for clinical trials [8,9] and hard-to-reach populations [10]. Hard-to-reach populations include those who are traditionally underrepresented in research studies, such as people from racial/ethnic minorities [11] and rural populations [12]. The feminine hygiene market has an estimated worth of US $35.4-$40 billion [13] and presents an exciting opportunity to recruit a large, diverse, and global population of individuals who menstruate in order to better understand the factors that may impact the hypothalamic-pituitary-ovarian axis. Similarly, the women’s health app market is estimated to be worth US $20.8 billion [14]. However, studies assessing the utility of these sources for recruitment to epidemiological research on ovulation and menstruation (OM) are lacking.

The ovulation and menstruation study (OM Global Health Study) recruited individuals from the mobile menstrual tracking app Clue and a boosted Facebook post by DivaCup (Diva International Inc.). Clue is a menstrual tracking app that was founded in 2013 by the Berlin-based company BioWink GmbH. It has over 12 million users from 190 different countries [15] and is considered to have 1 of the largest user bases worldwide among reproductive health apps [3]. Founded in 2001, Diva International Inc. is a Canadian menstrual care company that sells its menstrual cups in over 35 countries [16] and maintains engagement with its consumer base via its social media accounts, blog, and newsletter communications.

Previous research, such as that by Fenner et al [17], Wise et al [18], and Mahalingaiah et al [19], are examples of studies that recruited women through various digital modalities. Fenner et al [17] ran Facebook advertisements over a 5-month period targeting women in Victoria, Australia [17]. Wise et al [18] described recruitment for the Pregnancy Study Online (PRESTO) study over a 99-week period via internet ads, word of mouth, and flyers [18]. Lastly, in a cohort description paper among the first 10,000 participants in the Apple Women’s Health Study, multiple digital recruitment modalities were used [19].

The objective of this study was to assess the feasibility of recruiting a geographically diverse sample of women to an epidemiological study of OM health via digital recruitment. Feasibility and diversity were assessed via click and participation rates, geographic location, BMI, smoking status, and other demographic information about study participants.

## Methods

### OM Health Study

The OM Health Study is the umbrella study that includes the OM Health Pilot Study and the current OM Global Health Study. The OM Health Study was designed to advance our understanding of factors that promote menstrual health and awareness. Details regarding its health and lifestyle survey instrument and pilot launch within the United States have been previously described [20,21].

### Ethical Considerations

Participants provided informed consent to take part in the study. The web-based consent form included questions used to determine a participant’s interest in the current study, follow-up surveys, future studies, and contributing biospecimens. The parent study was approved by the Boston University Medical Campus Institutional Review Board (ID: IRB H-35075). De-identified study data were approved for analysis by the Harvard University Institutional Review Board (ID: IRB20-0638).

### Participant Recruitment

**Figure 1:**
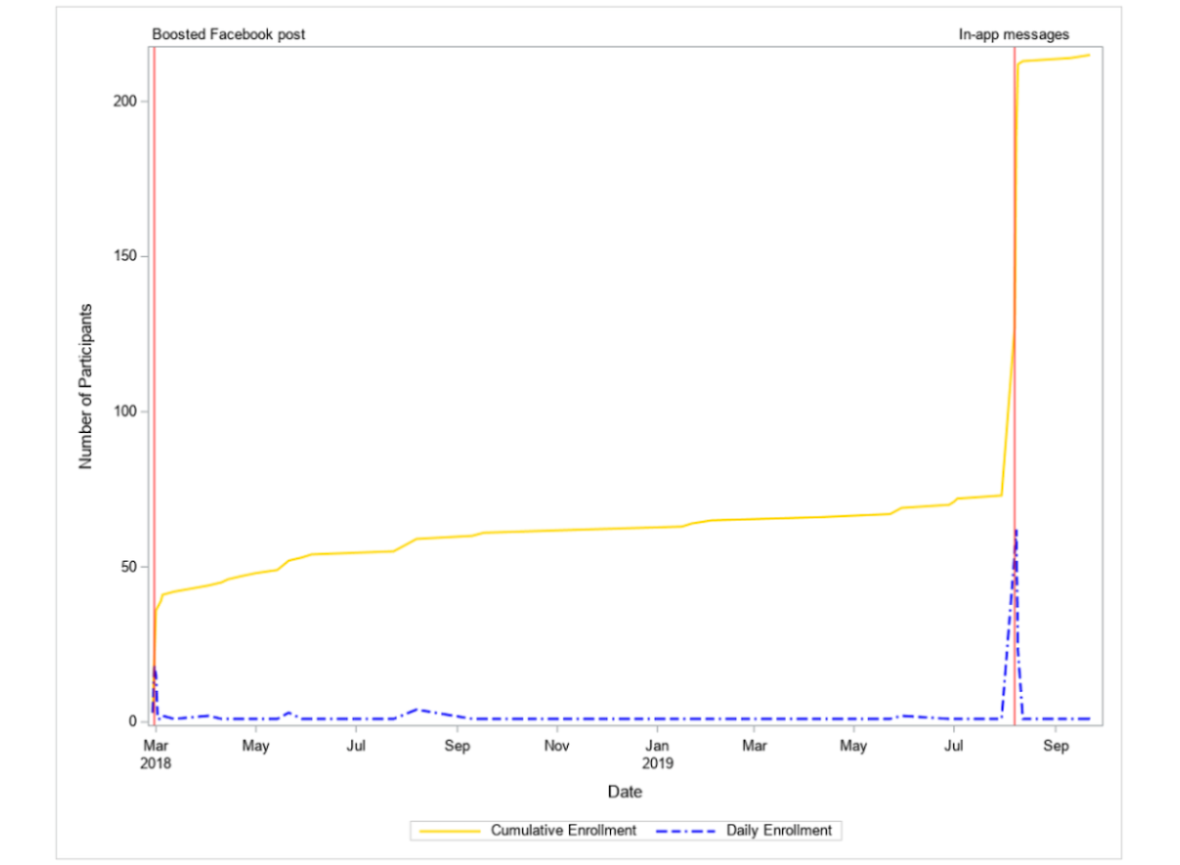
Timeline of digital recruitment. Note: Vertical red lines denote when active recruitment events (boosted Facebook post and in-app messages) occurred.

**Figure 2:**
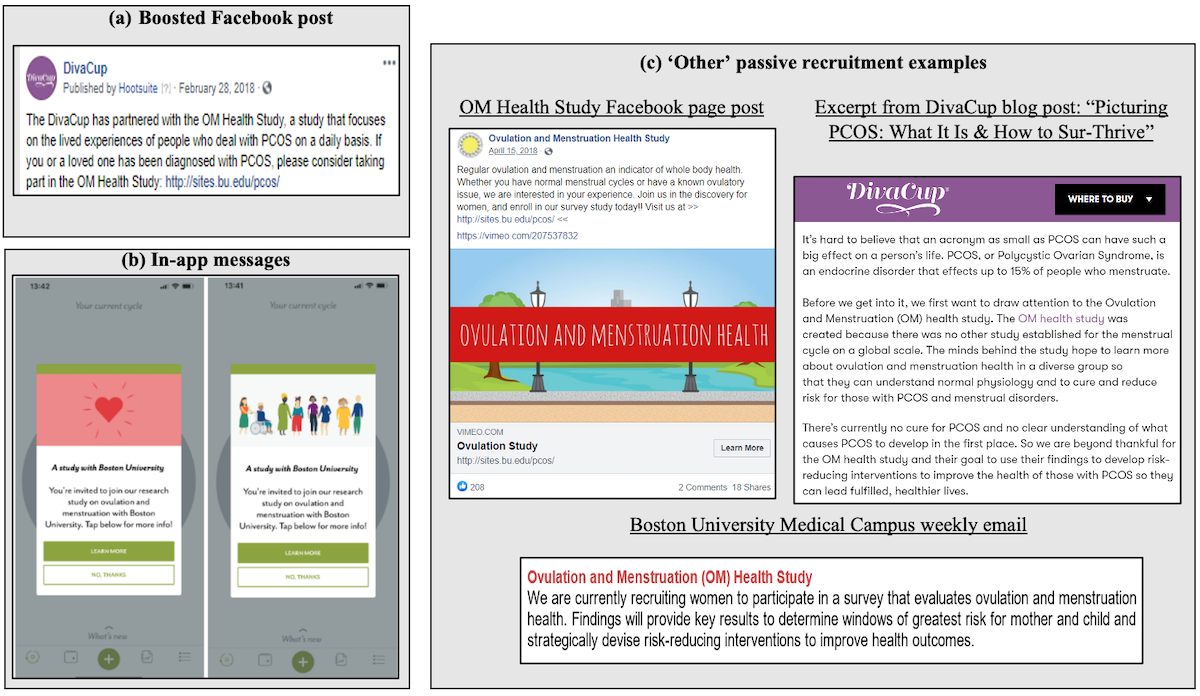
Digital recruitment materials: (a) boosted Facebook post, (b) in-app messages messaging, and (c) examples from other passive recruitment methods. PCOS: polycystic ovary syndrome.

A timeline of participant recruitment into the OM Global Health Study is displayed in Figure 1, and materials used to recruit participants are displayed in Figure 2. Participants were recruited online via 3 recruitment streams. The first was a DivaCup-boosted Facebook post including language related to a diagnosis of polycystic ovary syndrome (PCOS; “boosted Facebook post recruitment”) that was deployed on the DivaCup Facebook page on February 28, 2018 (Figure 2a). A boosted Facebook post is an ad that is created from posts on a Facebook page and is intended to create new user engagement and increase current user interactions [22]. The second stream comprised Clue in-app messages (“app recruitment”). In-app messages were sent to a subset of Clue users who were born after 2001, had the app language set to English, and had not received a survey from Clue within the past 60 days. Additionally, this subset comprised Clue users who opened the app during August 6-19, 2019 (Figure 2b). A third stream included other online passive recruitment study materials between February 27, 2018, and January 24, 2020 (Figure 2c). The other stream (“other passive recruitment”) consisted of posts on the OM Global Health Study’s social media accounts (LinkedIn, Twitter, and Facebook); DivaCup’s social media accounts, blog, or newsletter; or the Boston University Medical Campus weekly email blast that is sent to faculty, staff, and students. Passively recruited individuals were those who engaged with the other passive recruitment materials and those who may have encountered the study website on their own. To be eligible for the OM Global Health Study, individuals were required to be aged 18-44 years, not be currently pregnant, have no history of chemotherapy radiation or surgical menopause, and have a valid email address. The recruitment approach was split into 2 separate communications. Participants were given the link to the Research Electronic Data Capture (REDcap; Vanderbilt University)–administered survey and went through the consent and screener first. If they were eligible, they then received an email with an individualized link to the survey to continue their participation in the study.

### Recruitment and Participation Metrics

Click rate metrics were calculated to quantify the interest of potential participants for the OM Global Health Study website from the discrete recruitment events (ie, DivaCup and Clue). Click rate metrics were defined for the boosted DivaCup Facebook post and Clue in-app messages as the number of clicks per the number of ad views as reported by Facebook metrics and the number of clicks per in-app message views as reported by Braze customer engagement software for Clue, respectively.

Overall participation rates were calculated and defined as the number of completed surveys per number of completed consents and eligibility screeners. Survey completion was defined as finishing the pregnancy and birth history section of the OM Global Health Study questionnaire. The completion rate was defined as survey completion per number of initiated surveys. Initiated consents attributed to nonhuman engagement were excluded. We defined nonhuman engagement as survey forms that appeared to be initiated by a web app security scanner. Geographic, demographic, and health-related characteristics of participants who started the survey were evaluated using SAS 9.4 (SAS Institute).

## Results

### Recruitment and Enrollment

The click rate was 18.9% (19,700 clicks/104,000 ad views) via the Clue app and 1.6% via the boosted Facebook post (340 clicks/21,400 ad views). Overall, 215 individuals started the survey, of whom 140 (65.1%), 39 (18.1%), and 36 (16.8%) were recruited via the app, the boosted Facebook post, and other passive recruitment methods, respectively (Table 1). The first participant enrolled on February 27, 2018, and the last participant enrolled on September 22, 2019. Upticks in cumulative enrollment coincided with the deployment of the boosted Facebook post and in-app messages (Figure 1). The survey completion rate was 92.1% (198 completed per 215 started).

A total of 1466 consents and 856 eligibility screeners were completed, resulting in 444 consented and eligible individuals. Among those screened for eligibility (N=856), individuals were excluded if they were under 18 years of age (n=355, 41.5%), did not provide an email address (n=19, 2.2%), or were no longer menstruating (n=38, 4.4%). All those excluded because they were underage were recruited via the app. Of those who were recruited via the app (N=140) and provided a reason for nonparticipation in the eligibility screener (n=69, 49.3%), 64 (92.8%) reported the reason was their age and 52 (75.4%) specifically stated they were under the age of 18 years. The highest number of consents (n=2750, 46.2%) was initiated by an internet bot started on September 7, 2019; none of these progressed past the eligibility screener and were thus unable to start and complete the survey. Thus, the participation rate for the survey was 44.6% (198/444).

**Table 1:** Derivation of final study population and recruitment metrics by recruitment stream.

Metrics		Overall, N	Boosted Facebook post	In-app messages	Other passive recruitment
Date(s) of recruitment		February 27, 2018-January 24, 2020	February 28, 2018	August 6, 2019- August 19, 2019	February 27, 2018-January 24, 2020
Ad views, n (%)		125,400 (100)	21,400 (17.2)	104,000 (82.8)	N/A^a^
Ad clicks, n (%)		20,110 (100)	340 (1.7)	19,770 (98.3)	N/A
**Consent, n (%)**					
	Excluded: internet bot	2750 (100)	N/A	N/A	N/A
	Initiated	1755 (100)	74 (4.2)	1598 (91.1)	83 (4.7)
	Completed	1446 (100)	73 (5.1)	1293 (89.4)	80 (5.5)
**Eligibility screener, n (%)**					
	Excluded: under 18 years	355 (100)	0	355 (100)	0
	Excluded: no longer menstruating	38 (100)	20 (52.6)	2 (5.3)	16 (42.1)
	Excluded: email address not provided	19 (100)	0	19 (100)	0
	Completed	856 (100)	68 (7.9)	724 (84.6)	64 (7.5)
Eligible and consented		444 (100)	48 (10.8)	348 (78.4)	48 (10.8)
**OM^b^ Global Health Survey, n (%)**					
	Started	215 (100)	39 (18.1)	140 (65.1)	36 (16.8)
	Completed	198 (100)	37 (18.7)	128 (64.6)	33 (16.7)
**Recruitment metrics**					
	Click rate (clicks/views)	N/A	1.6% (340/21,400)	18.9% (19,700/104,000)	N/A
	Survey completion rate (#completed surveys/#eligible and consented)	44.6% (198/444)	77.1% (37/48)	36.8% (128/348)	68.8% (33/48)

^a^N/A: not applicable.

^b^OM: ovulation and menstruation.

### Participant Characteristics

The average age of the 215 participants who started the survey was 21.8 (SD 6.1) years. The average age of the participants was 28.8 (SD 6.3) years for the boosted Facebook post and 28.0 (SD 5.8) years for other passive recruitment. Due to the programming misspecification in the Clue app, only participants who reported that they were aged 18 years were notified about the study; thus, the average age was 18.3 (SD 1.1) years among Clue recruits (Table 2). Approximately, 119 (60%) of the 198 participants who started and completed the survey were 18 years old.

In terms of racial/ethnic diversity, 147 (70.7%) of the participants overall identified as non-Hispanic White, 35 (16.8%) more than 1 race/ethnicity, 10 (4.8%) Hispanic, 6 (2.9%) Asian, 4 (1.9%) Black, 4 (1.9%) Middle Eastern, and 2 (1.0%) other race/ethnicity. Overall, 123 (59.4%) of the participants had a high school education or less. In addition, 62 (29.8%) participants did not know their household income, 34 (16.4%) preferred not to answer, and the remaining majority (n=51, 24.5%) fell within the US $25,000-$74,999 income range, while 33 (15.9%) reported a household income of US $100,000 or more. Regarding health-related characteristics, the prevalence of smoking at least 100 cigarettes over their lifetime was 11.3% (n=22), and 31 (15.2%) and 49 (24.0%) of the participants were classified as overweight (BMI=25-29.9 kg/m^2^) and obese (BMI≥30.0 kg/m^2^), respectively. Overall, 104 (51.0%) reported having ever used a hormonal contraceptive, and 87 (43.3%) rated their health as good. There were no notable differences in demographic characteristics by survey completion status (Multimedia Appendix 1, Table S1). The prevalence of health characteristics is presented in Multimedia Appendix 1 (Table S2), and that of health characteristics among those 18 years old is presented in Multimedia Appendix 1 (Table S3). Of note, prevalence within the entire cohort versus those 18 years old was 22.7% and 3.4% for PCOS, 18.7% and 12.7% for gastroesophageal reflux disease (GERD), 21.2% and 23.7% for eating disorders, 3.5% and 3.4% for diabetes, 38.4% and 33.9% for depression, and 44.9% and 46.6% for anxiety, respectively.

**Table 2:** Demographic and health-related characteristics of participants, overall and by recruitment stream (N=215).

Characteristics		Overall (N=215)^a^	Boosted Facebook post (n=39)^b^	In-app messages (n=140)^c^	Other passive recruitment (n=36)^d^
Age (years), mean (SD; range)		21.8 (SD 6.1; 18-44)	28.8 (SD 6.3; 18-44)	18.3 (SD 1.1; 18-26)	28.0 (SD 5.8; 18-43)
**Residence^e^, n (%)**					
	Asia	8 (3.9)	0	6 (4.5)	2 (5.7)
	Australia	4 (1.9)	0	4 (3.0)	0
	Europe	46 (22.3)	2 (5.4)	44 (32.8)	0
	North America (outside the United States)	29 (14.1)	12 (32.4)	15 (11.2)	2 (5.7)
	South America	4 (1.9)	1 (2.7)	3 (2.2)	0
	United States	115 (55.8)	22 (59.5)	62 (46.3)	31 (88.6)
**Race/ethnicity, n (%)**					
	White (non-Hispanic)	147 (70.7)	32 (84.2)	91 (67.4)	24 (68.6)
	Latina/Hispanic	10 (4.8)	0	8 (5.9)	2 (5.7)
	Black/African American (non-Hispanic)	4 (1.9)	0	2 (1.5)	2 (5.7)
	Asian	6 (2.9)	0	4 (3.0)	2 (5.7)
	Middle Eastern	4 (1.9)	0	4 (3.0)	0
	Other race/ethnicity	2 (1.0)	0	2 (1.5)	0
	More than 1 race/ethnicity	35 (16.8)	6 (15.8)	24 (17.8)	5 (14.3)
**Educational attainment, n (%)**					
	High school graduate/General Educational Development (GED) or less	123 (59.4)	2 (5.3)	120 (89.6)	0 (0.0)
	Some college or 2-year degree	26 (12.6)	18 (47.4)	6 (4.5)	1 (2.9)
	4-year college graduate	33 (15.9)	13 (34.2)	6 (4.5)	14 (40.0)
	More than 4-year college degree	25 (12.1)	5 (13.2)	2 (1.5)	18 (51.4)
**Total annual household income (US $), n (%)**					
	Below 25,000	16 (7.7)	4 (10.5)	9 (6.7)	3 (8.6)
	25,000-49,999	24 (11.5)	8 (21.1)	9 (6.7)	7 (20.0)
	50,000-74,999	27 (13.0)	6 (15.8)	11 (8.2)	10 (28.6)
	75,000-99,999	12 (5.8)	2 (5.3)	7 (5.2)	3 (8.6)
	100,000 or mor	33 (15.9)	10 (26.3)	17 (12.6)	6 (17.1)
	Prefer not to answer	34 (16.4)	5 (13.2)	28 (20.7)	1 (2.9)
	Do not know	62 (29.8)	3 (7.9)	54 (40.0)	5 (14.3)
Smoked at least 100 cigarettes over lifetime, n (%)		22 (11.3)	8 (21.6)	10 (8.0)	4 (12.1)
**BMI (kg/m^2^), n (%)**					
	Underweight (<18.5)	17 (8.3)	0	17 (13.0)	0
	Normal weight (18.5-24.9)	107 (52.5)	4 (10.8)	84 (64.1)	19 (52.8)
	Overweight (25.0-29.9)	31 (15.2)	5 (13.5)	19 (14.5)	10 (27.8)
	Obese (≥30.0)	49 (24.0)	28 (75.7)	11 (8.4)	7 (19.4)
Hormonal contraceptives use ever, n (%)		104 (51.0)	35 (94.6)	38 (28.8)	31 (88.6)
**Participant’s rating of current health, n (%)**					
	Excellent	22 (11.0)	1 (2.7)	14 (10.8)	7 (20.6)
	Very good	66 (32.8)	11 (29.7)	40 (30.8)	15 (44.1)
	Good	87 (43.3)	19 (51.4)	58 (44.6)	10 (29.4)
	Fair	24 (11.9)	6 (16.2)	16 (12.3)	2 (5.9)
	Poor	2 (1.0)	0 (0.0)	2 (1.5)	0 (0.0)

^a^n (% complete): age, 215 (100%); residence, 206 (95.8%), race/ethnicity, 208 (96.7%); education, 207 (96.3%); income, 208 (96.7%); smoking status, 195 (90.7%); BMI, 204 (94.9%); hormonal contraceptive, 204 (94.9%); self-rated current health, 201 (93.5%); “complete” defined as pregnancy birth history complete in the questionnaire.

^b^n (% complete): age, 39 (100%); residence, 37 (94.9%); race/ethnicity, 38 (97.4%); education, 38 (97.4%); income, 38 (97.4%); smoking status, 37 (94.9%); BMI, 37 (94.9%); hormonal contraceptive, 37 (94.9%); self-rated current health, 37 (94.9%).

^c^n (% complete): age, 140 (100%); residence, 134 (95.7%); race/ethnicity, 135 (96.4%); education, 134 (95.7%); income, 135 (96.4%); smoking status, 125 (89.3%); BMI, 131 (93.6%); hormonal contraceptive, 132 (94.3%); self-rated current health, 130 (92.9%).

^d^n (% complete): age, 36 (100%); residence, 35 (97.2%); race/ethnicity, 35 (97.2%); education, 35 (97.2%); income, 35 (97.2%); smoking status, 33 (91.7%); BMI, 36 (100%); hormonal contraceptive, 35 (97.2%); self-rated current health, 34 (94.4%).

^e^“Residence” defined as country of birth reported and reporting not living in the United States.

### Geographic Diversity

**Figure 3:**
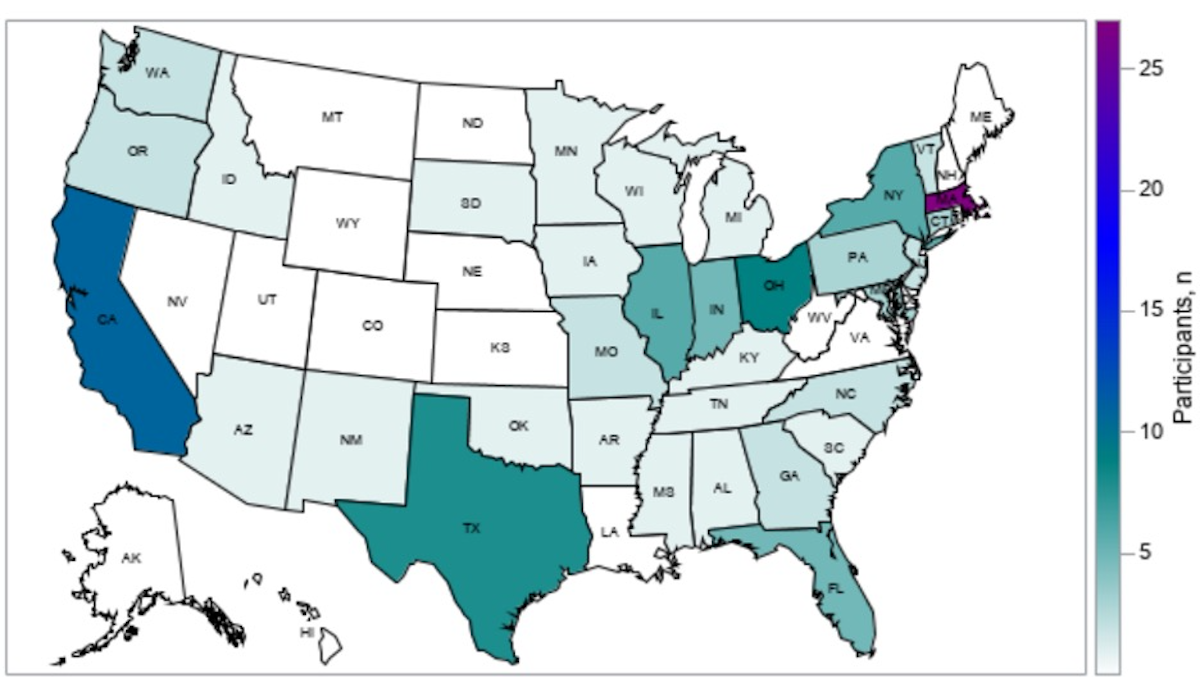
Geographic distribution of participants within the United States.

Overall, 115 (55.8%) of the 206 participants recruited via different modalities who completed the residence question resided in the United States, 46 (22.3%) in Europe, 29 (14.1%) outside the United States in North America, and approximately 16 (7.8%) in other parts of the world (Table 2). Recruitment via the Clue app (Germany) produced the most geographically diverse stream: 77 (57.5%) of the 134 participants recruited via the app who completed the residence question resided in North America, 44 (32.8%) in Europe, 6 (4.5%) in Asia, 4 (3.0%) in Australia, and 3 (2.2%) in South America. The boosted Facebook post through DivaCup (Canada) had the greatest proportion of participants who lived in parts of North America outside the United States (12/37, 32.4%). Most participants recruited via other passive recruitment methods (31/35, 88.6%) resided in the United States. Participants who were recruited within the United States, through all the modalities, resided in 33 different states, with over half located in 6 states: Massachusetts (n=27, 23.5%), California (n=11, 9.6%), Ohio (n=9, 7.8%), Texas (n=8, 7.0%), Illinois (n=6, 5.2%), and New York (n=6, 5.2%); see Figure 3.

### Technical Recruitment Errors

Two major errors occurred during the study period. One appeared to be due to an internet bot, and the second was a programming misspecification in the Clue app. Although these can be considered procedural breakdowns, screening of potential enrollees ensured eligibility requirements were met for those who did participate in the study. The internet bot appeared to result from a hacking attempt into the REDcap survey. This resulted in 2750 consent forms started on 1 day, with no further progression onto the study forms. These inputs were excluded from the study. The second procedural breakdown involved the age of those recruited via the Clue app. This was attributable to a programming misspecification for targeting the in-app message, which set the target birth year as greater than 2001 (instead of earlier than 2001). All these individuals were excluded from the study since they were under 18 years of age.

## Discussion

### Principal Findings

Digital recruitment via in-app messaging, a boosted Facebook post, and other passive recruitment methods was determined to be feasible. Previous studies have demonstrated that internet-based recruitment is cost-effective and typically increases cohort diversity, although it is limited geographically to specific regions, as seen in Fenner et al [17], Wise et al [18], and Mahaligaiah et al [19]. Moreover, previous studies have illustrated that diversity has increased in digital recruitment studies. Through the use of smartphone-based technology specifically, as was done in the MyHeart Counts study [23] and the Apple Women’s Health Study [19], our descriptive paper elaborated on the utility of these methods and, as detailed next, highlighted various limitations of each modality.

### Click and Participation Rates

We had a 44.6% overall participation rate, which is slightly above a previous health study that recruited women aged 16-25 years via a Facebook ad (24.5%) [17]. Although in-app recruitment had a higher click rate compared to the boosted Facebook post (19% vs 1.6%), the interpretation of these results was limited due to the programming misspecification that occurred upon initial release of the messaging. In addition, 75% of women from the in-app recruitment stream were not able to participate because they were under 18 years of age. Thus, this sample had overrepresentation of 18-year-olds. Future studies to assess age-related health risks will require additional stratification by age.

### Health Characteristics

We found that individuals who chose to participate across all 3 recruitment platforms had similar health characteristics, such as the BMI, smoking, and hormonal contraceptive use. These health-related findings likely reflect the large proportion of participants who were 18 years old, due to the programming misspecification. Thus, these findings may not be generalizable to the intended study population, which would have included larger proportions of individuals across the targeted age range of 18-45 years. Future studies should ensure that such problems do not arise in order to determine how digital platforms can be used to recruit individuals across a wider age range and with more variable health characteristics. Similarly, recruitment messaging targeting PCOS in the DivaCup post may oversample women with this disease [24]. Women using mobile menstrual tracking apps or those motivated to consume reproductive health information online may have differing goals, such as disease management, fertility enhancement, contraception, or health optimization, that may introduce selection bias that could impact measures of association derived from an epidemiological study using these forms of recruitment [5].

### Geographic Diversity

Diva International Inc. targets its advertisements to women primarily in Canada, the United States, Mexico, and Australia [25], while BioWink GmbH, which is based in Germany, is used in 190 countries worldwide [15]. The geographic reach of these companies is reflected in the geographic spread of our participants (ie, 44% were international). Additionally, participants recruited in the OM Global Health Study were more geographically diverse within the United States (33 states) compared to the OM Pilot Study (20 states) that relied primarily on in-person recruitment strategies [21]. Future epidemiological studies utilizing digital recruitment may benefit from partnering with similar companies with a wide geographic user base to target diverse participants for their research.

Additionally, in-app recruitment via Clue was able to not only reach a large audience (104,000 in-app message views) but also was rapid (in-app messaging occurred over a 2-week period) and inexpensive compared to traditional health research recruitment. Thus, in-app recruitment may be a cost-effective strategy that may enable health research in an increasingly strained funding environment.

### Limitations

Our study leveraged the consumer base of 2 major companies, Diva International Inc. and BioWink GmbH, and demonstrated the feasibility of using the platforms of these companies to recruit participants. Our results also demonstrated an opportunity to use mobile apps for recruitment of younger participants for research. Despite the widespread access to the internet and ownership of smartphones, some potential limitations of our study are the lack of ownership of electronic devices, such as smartphones and computers; digital illiteracy; and language barriers. Another major limitation of our study is the average age of those recruited via Clue due primarily to a programming misspecification. Although it would have been useful to see the age distribution of participants with targeting including those older than 18 years, our results show that younger women are interested in participating in research. Of those individuals who provided a reason for declining participation, 93% (n=64) stated that they were aged less than 18 years and 94% of ineligible participants (n=355) were deemed so due to age. Similarly, a prior study using the menstrual tracking app Flo to assess cycle variability found that approximately half of their participants were between the ages of 18 and 24 years [26]. These streams may present an opportunity for recruiting teenage women into health research. More importantly, this finding demonstrates that adequate study staff will be needed to reach out to participants and confirm eligibility, as was done by Fenner et al [17]. In addition to the protection of minors, it will be important to consider how demographic and health indicators are influenced by age. For example, younger generations tend to self-rate their health better than older generations [27].

Lastly, this study provides lessons for optimizing the use of digital tools in future research, including avoiding human and nonhuman sources of procedural breakdowns and improving information technology management. With traditional in-person recruitment, errors can be more easily identified than with digital recruitment. For example, it is simple for research assistants working in a clinic to identify recruitment errors, such as approaching participants outside a specified age and correcting them in real time. Additionally, hacking attempts by internet bots are specific to a digital space. Public surveys, especially those distributed via social media, are susceptible to hacking attempts; thus, implementing the “endCAPTCHA” module in REDcap may prevent this issue as it would provide insurance against bots inputting data as it serves as a human verification tool. To increase the utility of recruitment in the digital space, we recommend careful assessment of the programming process in real time in order to quickly identify and correct any anomalies in the resulting data. Server capacities also introduced limitations to our study. Initially, the consent form took up to 1-2 minutes to load, which may have resulted in participant drop-off before women were able to complete the eligibility and screening forms. Not only did the consent form load slowly, but when opening the link from an email, loading time varied, which may have also contributed to the low number of women enrolling in the survey. This may also have resulted in the exclusion of individuals with low income who tend to rely on a free or inexpensive Wi-Fi connection, exacerbating the underrepresentation of low-income women in health research [28]. Another issue would be language barriers in accessing our survey. As 1 of the inclusion criteria was being able to read English, we may have limited the scope of individuals who may have otherwise participated in this study.

### Conclusion

The broad scope of digital recruitment in this study allowed for more geographically, racially/ethnically, and age diverse participants than our OM Pilot Study, which relied on primarily in-person recruitment strategies. This study demonstrated the utility of a digital recruitment approach to successfully recruit participants worldwide. In-app recruitment resulted in the greatest number of surveys completed and contributed the most geographic diversity. Recruitment via the boosted Facebook post and other passive recruitment methods helped us further target specific audiences, such as those interested in participating due to general interest in women’s health. Procedural breakdowns also demonstrated the possible challenges in engaging with various digital platforms during research studies, while highlighting an opportunity to engage premenarchal- and menarchal-aged women in health research via app recruitment. Our findings and limitations may be useful to inform future epidemiological studies implementing digital recruitment.

## Appendix

Multimedia Appendix 1 Demographic characteristics by survey completion status and the prevalence of health characteristics.

## Abbreviations

OM: ovulation and menstruation
PCOS: polycystic ovary syndrome
