# Supplementary material for: Digital Global Recruitment for Women’s Health Research: Cross-sectional Study
Source: JMIR Form Res. 2022 Sep 14;6(9):e39046. doi: 10.2196/39046 (PMC9520381; doi:10.2196/39046)
Supplement: Multimedia Appendix 1 [file formative_v6i9e39046_app1.docx]

**Table S1.** Demographics of participants recruited, by survey completion status (N=215).

| Demographics | | Complete  (n=198)^a^ | Incomplete  (n=17)^b^ |
| --- | --- | --- | --- |
| Age (years), mean (SD; range) | | 21.9 (SD 6.1; 18-44) | 20.5 (SD 5.4; 18-35) |
| **Residence^c^, n (%)** | | | |
|  | Asia | 6 (3.1) | 2 (14.3) |
|  | Australia | 4 (2.1) | 0 |
|  | Europe | 42 (21.9) | 4 (28.6) |
|  | North America (outside US) | 29 (15.1) | 0 |
|  | South America | 4 (2.1) | 0 |
|  | United States | 107 (55.7) | 8 (57.1) |
| **Race/ethnicity, n (%)** | | | |
|  | White (non-Hispanic) | 138 (69.7) | 9 (90.0) |
|  | Latina/Hispanic | 10 (5.1) | 0 |
|  | Black/African American (non-Hispanic) | 4 (2.0) | 0 |
|  | Asian | 5 (2.5) | 1 (10.0) |
|  | Middle Eastern | 4 (2.0) | 0 |
|  | Other race/ethnicity | 2 (1.0) | 0 |
|  | More than 1 race/ethnicity | 35 (17.7) | 0 |
| **Educational attainment, n (%)** | | | |
|  | High school graduate/General Educational Development (GED) or less | 116 (58.9) | 7 (70.0) |
|  | Some college or 2-year degree | 25 (12.7) | 1 (10.0) |
|  | 4-year college graduate | 32 (16.2) | 1 (10.0) |
|  | More than 4-year college degree | 24 (12.2) | 1 (10.0) |
| **Total annual household income (US $), n (%)** | | | |
|  | Below 25,000 | 16 (8.1) | 0 (0.0) |
|  | 25,000-49,999 | 20 (10.1) | 4 (40.0) |
|  | 50,000-74,999 | 26 (13.1) | 1 (10.0) |
|  | 75,000-99,999 | 11 (5.6) | 1 (10.0) |
|  | 100,000 or more | 33 (16.7) | 0 (0.0) |
|  | Prefer not to answer | 33 (16.7) | 1 (10.0) |
|  | Do not know | 59 (29.8) | 3 (30.0) |

^a^“Complete” defined as pregnancy birth history complete in the questionnaire. n (% complete): age, 198 (100); residence, 192 (97.0); race/ethnicity, 198 (100); education, 197 (95.5); income, 198 (100).

^b^n (% complete): age, 17 (100); residence, 3 (82.4); race/ethnicity, 10 (58.8); education, 10 (58.8); income, 10 (58.8).

^c^“Residence” defined as country of birth reported and reporting not living in the United States.

**Table S2.** Ovulation and menstruation global general health and reproductive disorders for all participants with completed surveys, by recruitment method (N=198).

| Disorders | | Complete^a^ (n=198), n (%) | Boosted Facebook post (n=37), n (%) | In-app messages (n=128), n (%) | Other passive recruitment (n=33), n (%) |
| --- | --- | --- | --- | --- | --- |
| **Polycystic ovary syndrome (PCOS)** | | | | |  |
|  | Yes | 45 (22.7) | 30 (81.1) | 3 (2.3) | 12 (36.4) |
| **Gastroesophageal reflux disease (GERD)** | | | | |  |
|  | Yes | 37 (18.7) | 13 (35.1) | 18 (14.1) | 6 (18.2) |
|  | Missing | 2 (1.0) | 0 (0.0) | 2 (1.6) | 0 (0.0) |
| **Have you ever had an eating disorder?** | | | | |  |
|  | Yes | 42 (21.2) | 6 (16.2) | 32 (25.0) | 4 (12.1) |
|  | Missing | 1 (0.5) | 0 (0.0) | 1 (0.8) | 0 (0.0) |
| **High blood pressure (not during pregnancy)** | | | | |  |
|  | Yes | 11 (5.6) | 2 (5.4) | 5 (3.9) | 4 (12.1) |
| **High cholesterol** | | | | |  |
|  | Yes | 13 (6.6) | 6 (16.2) | 6 (4.7) | 1 (3.0) |
| **Diabetes (not during pregnancy)** | | | | |  |
|  | Yes | 7 (3.5) | 1 (2.7) | 5 (3.9) | 1 (3.0) |
| **Nonalcoholic fatty liver disease** | | | | |  |
|  | Yes | 3 (1.5) | 2 (5.4) | 0 (0.0) | 1 (3.0) |
|  | Do not know | 15 (7.6) | 3 (8.1) | 8 (6.2) | 4 (12.1) |
|  | Missing | 1 (0.5) | 0 (0.0) | 1 (0.8) | 0 (0.0) |
| **Thyroid disease** | | | | |  |
|  | Yes | 7 (3.5) | 2 (5.4) | 2 (1.6) | 3 (9.1) |
|  | Do not know | 10 (5.1) | 1 (2.7) | 8 (6.2) | 1 (3.0) |
|  | Missing | 2 (1.0) | 0 (0.0) | 2 (1.6) | 0 (0.0) |
| **Excess prolactin** | | | | |  |
|  | Yes | 3 (1.5) | 2 (5.4) | 0 (0.0) | 1 (3.0) |
|  | Do not know | 9 (4.5) | 3 (8.1) | 4 (3.1) | 2 (6.1) |
|  | Missing | 2 (1.0) | 0 (0.0) | 2 (1.6) | 0 (0.0) |
| **Sleep apnea** | | | | |  |
|  | Yes | 5 (2.5) | 1 (2.7) | 4 (3.1) | 0 (0.0) |
|  | Missing | 1 (0.5) | 0 (0.0) | 1 (0.8) | 0 (0.0) |
| **Posttraumatic stress disorder (PTSD)** | | | | |  |
|  | Yes | 20 (10.1) | 7 (18.9) | 9 (7.0) | 4 (12.1) |
|  | Missing | 1 (0.5) | 0 (0.0) | 1 (0.8) | 0 (0.0) |
| **Chronic fatigue syndrome** | | | | |  |
|  | Yes | 5 (2.5) | 0 (0.0) | 3 (2.3) | 2 (6.1) |
|  | Missing | 4 (2.0) | 1 (2.7) | 1 (0.8) | 2 (6.1) |
| **Seizure disorder** | | | | |  |
|  | Yes | 1 (0.5) | 0 (0.0) | 0 (0.0) | 1 (3.0) |
|  | Missing | 1 (0.5) | 0 (0.0) | 1 (0.8) | 0 (0.0) |
| **Depression** | | | | |  |
|  | Yes | 76 (38.4) | 20 (54.1) | 43 (33.6) | 13 (39.4) |
|  | Missing | 1 (0.5) | 0 (0.0) | 1 (0.8) | 0 (0.0) |
| **Anxiety or panic disorder** | | | | |  |
|  | Yes | 89 (44.9) | 15 (40.5) | 62 (48.4) | 12 (36.4) |
|  | Missing | 1 (0.5) | 0 (0.0) | 1 (0.8) | 0 (0.0) |

^a^“Complete” defined as pregnancy birth history complete in the questionnaire. Missing amounts were reported. Categories with no missing reported did not have any missing responses.

**Table S3.** Ovulation and menstruation global general health and reproductive disorders for 18-year-olds with completed surveys, by recruitment method (N=118).

| Disorders | | Complete^a^ (n=118), n (%) | Boosted Facebook post (n=2), n (%) | In-app messages (n=116), n (%) |
| --- | --- | --- | --- | --- |
| **Polycystic ovary syndrome (PCOS)** | | | | |
|  | Yes | 4 (3.4) | 2 (100.0) | 2 (1.7) |
| **Gastroesophageal reflux disease (GERD)** | | | | |
|  | Yes | 15 (12.7) | 0 (0.0) | 15 (12.9) |
|  | Missing | 2 (1.7) | 0 (0.0) | 2 (1.7) |
| **Eating disorder** | | | |  |
|  | Yes | 28 (23.7) | 0 (0.0) | 28 (24.1) |
|  | Missing | 1 (0.8) | 0 (0.0) | 1 (0.9) |
| **High blood pressure (not during pregnancy)** | | | |  |
|  | Yes | 5 (4.2) | 0 (0.0) | 5 (4.3) |
| **High cholesterol** | | | |  |
|  | Yes | 5 (4.2) | 0 (0.0) | 5 (4.3) |
| **Diabetes (not during pregnancy)** | | | | |
|  | Yes | 4 (3.4) | 0 (0.0) | 4 (3.4) |
| **Nonalcoholic fatty liver disease** | | | | |
|  | Yes | 0 (0.0) | 0 (0.0) | 0 (0.0) |
|  | Do not know | 8 (6.8) | 1 (50.0) | 7 (6.0) |
|  | Missing | 1 (0.8) | 0 (0.0) | 1 (0.9) |
| **Thyroid disease** | | | | |
|  | Yes | 2 (1.7) | 0 (0.0) | 2 (1.7) |
|  | Do not know | 7 (5.9) | 0 (0.0) | 7 (6.0) |
|  | Missing | 2 (1.7) | 0 (0.0) | 2 (1.7) |
| **Excess prolactin** | | | |  |
|  | Yes | 0 (0.0) | 0 (0.0) | 0 (0.0) |
|  | Do not know | 3 (2.5) | 0 (0.0) | 3 (2.6) |
|  | Missing | 1 (0.8) | 0 (0.0) | 1 (0.9) |
| **Sleep apnea** | | | |  |
|  | Yes | 5 (2.5) | 0 (0.0) | 3 (2.6) |
|  | Missing | 1 (0.5) | 0 (0.0) | 1 (0.9) |
| **Posttraumatic stress disorder (PTSD)** | | | | |
|  | Yes | 3 (2.5) | 0 (0.0) | 5 (4.3) |
|  | Missing | 1 (0.8) | 0 (0.0) | 1 (0.9) |
| **Chronic fatigue syndrome** | | | |  |
|  | Yes | 5 (4.2) | 0 (0.0) | 2 (1.7) |
|  | Missing | 1 (0.8) | 0 (0.0) | 1 (0.9) |
| **Seizure disorder** | | | |  |
|  | Yes | 0 (0.0) | 0 (0.0) | 0 (0.0) |
|  | Missing | 1 (0.8) | 0 (0.0) | 1 (0.9) |
| **Depression** | | | |  |
|  | Yes | 40 (33.9) | 1 (50.0) | 39 (33.6) |
|  | Missing | 1 (0.8) | 0 (0.0) | 1 (0.9) |
| **Anxiety or panic disorder** | | | |  |
|  | Yes | 55 (46.6) | 1 (50.0) | 54 (46.6) |
|  | Missing | 1 (0.8) | 0 (0.0) | 1 (0.9) |

^a^“Complete” defined as pregnancy birth history complete in the questionnaire. Missing defined as those who did not respond to the question.
